# Molecular profiling of non-small cell lung cancer

**DOI:** 10.1371/journal.pone.0236580

**Published:** 2020-08-05

**Authors:** Marika L. Forsythe, Akram Alwithenani, Drew Bethune, Mathieu Castonguay, Arik Drucker, Gordon Flowerdew, Daniel French, John Fris, Wenda Greer, Harry Henteleff, Mary MacNeil, Paola Marignani, Wojciech Morzycki, Madelaine Plourde, Stephanie Snow, Zhaolin Xu

**Affiliations:** 1 Department of Pathology, Faculty of Medicine, Dalhousie University Halifax, Halifax, Nova Scotia, Canada; 2 Department of Laboratory Medicine, Faculty of Applied Medical Sciences, Umm Al-Qura University Makkah, Makkah, Saudi Arabia; 3 Department of Surgery, Faculty of Medicine, Dalhousie University, Halifax, Nova Scotia, Canada; 4 Division of Medical Oncology, Faculty of Medicine, Dalhousie University, Halifax, Nova Scotia, Canada; 5 Department of Epidemiology, Faculty of Medicine, Dalhousie University, Halifax, Nova Scotia, Canada; 6 Department of Biochemistry and Molecular Biology, Faculty of Medicine, Dalhousie University, Halifax, Nova Scotia, Canada; Universidade do Algarve Departamento de Ciencias Biomedicas e Medicina, PORTUGAL

## Abstract

Lung cancer is generally treated with conventional therapies, including chemotherapy and radiation. These methods, however, are not specific to cancer cells and instead attack every cell present, including normal cells. Personalized therapies provide more efficient treatment options as they target the individual’s genetic makeup. The goal of this study was to identify the frequency of causal genetic mutations across a variety of lung cancer subtypes in the earlier stages. 833 samples of non-small cell lung cancer from 799 patients who received resection of their lung cancer, were selected for molecular analysis of six known mutations, including *EGFR*, *KRAS*, *BRAF*, *PIK3CA*, *HER2 and ALK*. A SNaPshot assay was used for point mutations and fragment analysis searched for insertions and deletions. *ALK* was evaluated by IHC +/- FISH. Statistical analysis was performed to determine correlations between molecular and clinical/pathological patient data. None of the tested variants were identified in most (66.15%) of cases. The observed frequencies among the total samples vs. only the adenocarcinoma cases were notable different, with the highest frequency being the *KRAS* mutation (24.49% vs. 35.55%), followed by *EGFR* (6.96% vs. 10.23%), *PIK3CA* (1.20% vs. 0.9%), *BRAF* (1.08% vs. 1.62%), *ALK* (0.12% vs. 0.18%), while the lowest was the *HER2* mutation (0% for both). The statistical analysis yielded correlations between presence of a mutation with gender, cancer type, vascular invasion and smoking history. The outcome of this study will provide data that helps stratify patient prognosis and supports development of more precise treatments, resulting in improved outcomes for future lung cancer patients.

## Introduction

Lung cancer is one of the most frequent causes of cancer-related deaths among men and women in Canada, the United States and many other countries worldwide. One in eight cancer occurrences is lung cancer, which leads to the death of 1.1 million people each year with only 7–18% five-year survival rate [1, 2]. The three major histologic types of NSCLC include adenocarcinoma, squamous cell carcinoma, and large cell carcinoma. Adenocarcinoma is the most common type of lung carcinomas, accounting for about 40% of cases, and is also the most diverse in histological patterns. Proper diagnosis of adenocarcinoma is sometimes difficult among small biopsies in the absence of the physical structure [3, 4]. The mutations most frequently reported in adenocarcinoma occur in the *KRAS* and *EGFR* genes [5]. The genetic variations in squamous cell carcinomas are distinct, and no targeted therapies directed against its genetic alterations [6]. Approximately 80% of squamous cell tumours exhibit overexpression of *EGFR*, although mutations are rarely identified in the gene. Approximately 30% of these tumours overexpress the *HER2* gene [5].

The majority of lung cancer patients present at advanced stages of the disease [7]. Previous treatment options for advanced stage lung cancer were limited to chemotherapy and radiation, showing a response rate of around 20%-30%. Newer targeted therapies based on the genotype of a patient’s cancer cells allows targeting a specific active kinase and a much higher response rate for those cases, reaching 75% with improved quality of life [8]. For tumours identified to not carry a mutation leading to drug responsiveness, early identification could lead to earlier surgical resection, however other treatments such as adjuvant chemotherapy would often be required either immediately following resection or in later stages of the disease when surgery was no longer an option [9].

Roughly 60% of adenocarcinoma cases contain a driver mutation, initiating the process. The most common driver mutations are found in *KRAS* (V-Ki-ras2 Kirsten rat sarcoma viral oncogene homolog; 25%), *EGFR* (epidermal growth factor receptor; 23%), *ALK* (anaplastic lymphoma kinase; 6%), *PIK3CA* (phosphoinositide-3-kinase, catalytic, alpha polypeptide; 3%), *BRAF* (v-Raf murine sarcoma viral oncogene homolog B1; 3%), and *HER2* (human epidermal growth factor receptor 2; 1%) [8, 9]. The resulting aberrant gene products are involved in signalling pathways associated with cell proliferation and cell survival.

The development of therapies that target specific mutations in lung cancer has revolutionized the therapy for patients who harbour a targetable mutation. Treating most patients with *EGFR* and *ALK* mutations with targeted tyrosine kinase inhibitors (TKIs) is standard of care and has been proven to be better tolerated and more efficacious than cytotoxic chemotherapy as initial therapy [10–12]. There are now multiple drugs available for patients with targetable *EGFR* and *ALK* mutations, and better understanding of how tumours become resistant to initial lines of therapy has allowed for new drug development that circumvents resistance such that many patients can be treated with multiple TKIs in sequence before requiring chemotherapy [13, 14]. For example, more than half of patients with a targetable *EGFR* mutation who receive initial therapy with a first- or second-generation *EGFR* TKI (gefitinib, erlotinib or afatinib) will become resistant by developing a new T790M point mutation [14–17]. The third generation *EGFR* TKI osimertinib has activity in this patient group and has been established as the standard second line therapy for a patient with an acquired T790M mutation. Further, there is data that suggests that starting with osimertinib in the first line setting is another good option, and patients treated with first line osimertinib do not seem to develop the T790M mutation [18–20].

In addition to understanding and targeting resistance mutations, some targeted TKIs have better activity in patients with CNS metastasis of their lung cancer. This is due to either improved penetration of the drug into the CNS or medications that are effective at very low concentrations, and hence work for patients with brain metastasis when only a small amount of the drug crosses the blood brain barrier. This has translated into better outcomes for patients who develop progression on an initial TKI with brain metastasis, allowing some patients to avoid or delay brain radiation [21–23]. Furthermore, some TKIs have been described as “CNS protective” meaning that patients receiving those TKIs have a lower rate of developing new brain metastasis [24]. There are multiple other driver mutations that have been identified in lung cancer that have effective targeted therapies, including *ROS1*, *BRAF*, and *NTRK* mutations to name a few [25, 26].

Previously published studies have included mainly advanced stage lung cancer patients, and little is known about mutations in patients with early stage of the disease and clinical outcome after surgical treatment. Therefore, this study involved the mutational status in a group of mainly early stage lung cancer patients treated with surgical resection of the tumours. All samples were submitted with patient consent to access their history, making it possible to do clinical and pathological correlations.

This study hypothesized that the correlations determined between molecular and clinical/pathological data in the cohort will match those observed in other published studies for different populations across the world. In order to do the correlations, an appropriate tumour sample was selected for each case and molecular profiling was performed on the samples to determine the frequency of mutations. In addition, clinical and pathological features of the patients were collected. Such correlations provide the preliminary data that could be used for future research, both in the development of personalized treatments for new mutations or improvement of treatment options for previously treated mutations.

## Materials and methods

### Case selection and clinical data collection

A total of 799 surgically resected non-small cell lung cancer cases with sufficient molecular data were collected from 2005 to 2016 at the Queen Elizabeth II Health Sciences Centre in Halifax, Canada for the study. Among those 799 cases, 28 had more than one primary lung tumours, bringing the total number of samples used in this study to 833. Diagnosis of lung cancer subtype for each case was achieved by morphology assessment and immunohistochemistry (TTF-1, Napsin A, P40/P63, CH5/6, CK7, CK20). All participants signed an informed written consent and both the consent and study are approved by Nova Scotia Health Authority REB (#1011704). The written consent was signed before or after their surgery for lung cancer and agreed to contribute their tumour tissue to the QEII Lung Tumour Bank (certified by Canadian Tissue Repository Network) for any future lung cancer research. The participants were from the lung tumour bank sequentially. There were no minors in this cohort.

### Mutation analysis

#### Analysis for ALK rearrangements

*ALK* mutations reported in NSCLC are primarily chromosomal inversions or translocations that result in an *ALK-EML4* fusion gene and elevated expression of abnormal *ALK* fusion protein. All cases were initially screened for high levels of *ALK* expression using immunohistochemistry (IHC) with *ALK* 5A4 antibody from Leica on Ventana BenchMark ULTRA IHC staining system; equivocal or positive cases were confirmed with fluorescent in situ hybridization (FISH) using the Vysis^™^ *ALK* break-apart probe (Abbott Molecular).

#### Analysis for point mutations

A SNaPshot (multiplexed primer extension) assay was developed to detect recurring point mutations at seven nucleotide positions [27]. These include the c.2573T>G (p.Leu858Arg, or L858R) and c.2369C>T (p.Thr790Met, or T790M) mutations in *EGFR* (NM_005228.4), the c.34G>T (p.Gly12Cys) and c.35G>T (p.Gly12Val) mutations in *KRAS* (NM_004985.4), the c.1624G>A (p.Glu542Lys, or E542K) and c.1633G>A (p.Glu545Lys, or E545K) mutations in *PIK3CA* (NM_006218.3), and the c.1799T>A (p.Val600Glu, or V600E) mutation in *BRAF* (NM_004333.5). Due to its multiplexed nature, the assay can detect up to ten-point mutations simultaneously from a very small amount of DNA. This is critical considering the amount of tissue available for testing is often limited [28]. Briefly, DNA was extracted from tumour tissue on five unstained glass slides with 20 μm sections. Tumour was identified and indicated on the slides by an anatomical pathologist based on H&E staining of one section. Tumour was scraped from the slides into a 1.5 mL microcentrifuge tube with 300 μL QIAGEN ATL buffer and 2 mg/mL Proteinase K, incubated overnight at 65°C, and then extracted using the Magna Pure Compact automated DNA extraction platform (Roche). The custom SNaPshot assay, containing PCR primers and extension primers, was used according to the manufacturer’s instructions (ABI PRISM SNaPshot Multiplex Kit cat#4323151) and products were resolved on an ABI 3130XL capillary sequencer (Applied Biosystems). The dNTPs used were dideoxy nucleotides, each labelled with a different fluorescence for analysis [29]. This assay requires 20 ng of input DNA and is sensitive enough to detect the mutant alleles in tumour cells that comprise as low as 1% to 10% of total nucleated cells.

#### Analysis for insertions and deletions

A fragment analysis sizing assay was used to detect small recurring deletions in exon 19 of *EGFR* and insertions in exon 20 of *EGFR* and exon 20 of *HER2*. This is a multiplexed assay that uses differentially labelled fluorescent PCR primers specific for regions that flank the deletion/insertion sites to generate amplicons that are sized and detected using a capillary sequencer as described above. This assay is sensitive enough to detect as low as 1% to 10% mutant alleles in a background of wild-type alleles.

### Statistical analysis

Statistical analysis was performed using the SAS program. The data was analysed to determine if there were correlations between the molecular data and pathological/clinical data of the patients. Mutation was cross-tabulated by age, gender, cancer type, pleural invasion, vascular invasion, lymphatic invasion, lymph node invasion, tumour stage (based on AJCC 8^th^ edition staging), smoking history and 5-year survival. The contribution of each cell (Observed-Expected)^2^/Expected to the overall chi-square statistic indicated which cells in the table provided evidence of an association. A value greater than 3.841 can be used as a conservative cut-off for statistical significance when the expected frequency is at least 5, and as a rough guide when the expected frequency is less than 5. Overall presence of an association was assessed using the chi-square test. Since the statistical significance of this test is exaggerated when some of the expected frequencies are less than 5, the calculated p-values provide only a rough indication of the strength of evidence for an association.

Cox proportional hazards analysis was used to investigate if overall patient survival, not just 5-year survival, was related to mutation.

## Results

**Fig 1:**
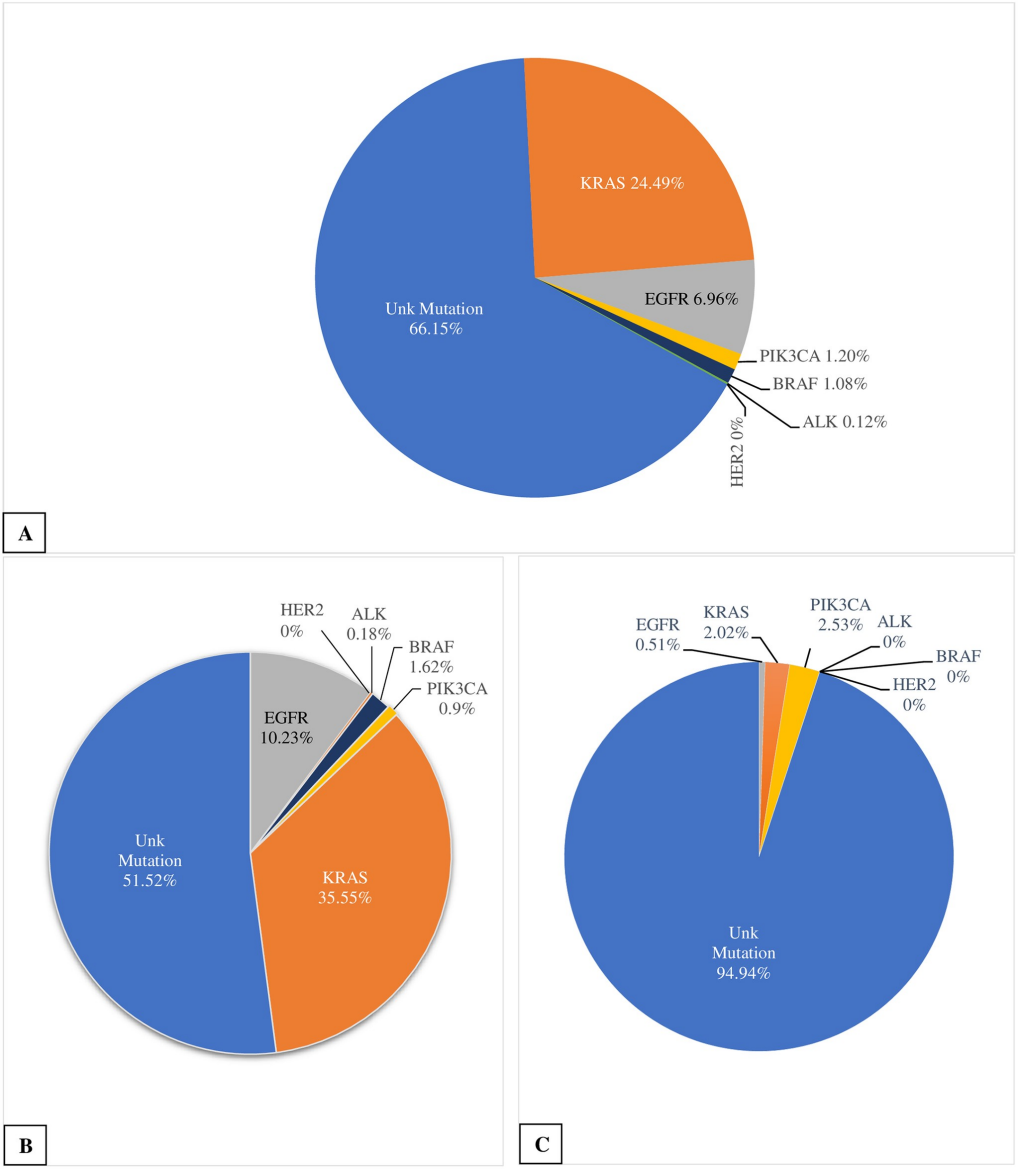
Mutation frequencies. A. Frequency of the mutations within the 833 samples tested. B. Frequency of the mutations with the 557 adenocarcinoma samples tested. C. Frequency of the mutations with the 198 squamous cell carcinoma samples tested.

Among the 799 cases available, 392 were males and 407 females with the age ranging from 34 to 90 years. Other demographic data concerning the patients are listed in Table 1. There were 557 samples of adenocarcinoma (66.87%), 198 samples of squamous cell carcinoma (23.77%), 53 samples of large cell carcinoma (6.36%), and among the lesser common types there were 12 samples of pleomorphic carcinoma (1.44%) and 13 samples of carcinoid (1.56%). Molecular genotyping of the tumour tissue showed that overall, about 66.15% of the cases had no mutations in the genes studied. In the remaining cases, *KRAS* exhibited were the most frequent (24.49%), followed by the *EGFR* (6.96%), *PIK3CA* (1.2%), *BRAF* (1.08%), and *ALK* gene rearrangement (0.12%), while *HER2* mutations were not present at all in this cohort. Among 58 cases with *EGFR* mutations 30 had an exon 19 deletion (3.6% of total), 25 had an exon 21 L858R mutation (3.0%), and 3 had an exon 20 insertion (0.36%). In 10 cases with *PIK3CA* mutations, 4 were *PIK3CA* E452K (0.48% of total) and 6 were *PIK3CA* E545K (0.72%). There was also one case which exhibited both a *KRAS* and *PIK3CA* mutation. When only the adenocarcinoma cases were examined, *KRAS* exhibited the highest rate of mutation (35.55%). It was followed by the *EGF*R mutation (10.23%), *BRAF* mutation (1.62%), *PIK3CA* mutation (0.9%), and *ALK* gene rearrangement (0.18%). The remaining 51.52% of the cases had no mutations in the genes studied. Among squamous cell carcinoma cases, mutations of the genes only accounted for 5% with *PIK3CA* exhibited the highest rate of mutation (2.53%). It was followed by the *KRAS* mutation (2.02%) and *EGFR* mutation (0.51%). The other mutations were not seen (Fig 1).

**Table 1:** Demographic data of the 833 samples from the 799 cases.

*Parameter*	*Total*
**Gender**	
Male	392 (49.1%)
Female	407 (50.9%)
**Age**	
39–59	171 (21.4%)
60–74	460 (57.6%)
75–90	168 (21.0%)
**Smoking History**	
Never Smoked	55 (6.9%)
Ever Smoked	744 (93.1%)
**Tumour Type**^a^	
Adenocarcinoma	557 (66.9%)
Squamous cell carcinoma	198 (23.8%)
Large cell carcinoma (including large cell neuroendocrine carcinoma)	53 (6.4%)
Pleomorphic carcinoma	12 (1.4%)
Carcinoid	13 (1.5%)
**Tumour Stage**^a^	
I (a and b)	437 (52.5%)
II (a and b)	217 (26.1%)
III (a and b)	165 (19.8%)
IV	7 (0.8%)
Unknown	7 (0.8%)
**Surgery**	
Lobectomy	634 (79.4%)
Pneumonectomy	73 (9.1%)
Wedge resection	73 (9.1%)
Segmentectomy	16 (2.0%)
Lymph node excision	2 (0.3%)
Bi-lobectomy	1 (0.1%)
**Five-year survival (mortality)**	
Median number	100 months

^a^Used the total 833 tumour samples to calculate.

Most of the patients had early stage disease at the time of surgical treatment with stage I 52.5% and stage II 26.1% (Table 1). Tables 2–4 show the observed frequency in each cell, the expected frequency under the null hypothesis of no association, and the contribution of each cell to the overall chi-square statistic. Given that the degrees of freedom of the overall chi-square statistic is equal to the (R-1)(C-1) where R and C are the numbers of rows and columns, respectively, the contribution of each cell the overall chi-square statistic is a chi-square with slightly less than one degree of freedom. A value of more than 3.841 (the 95^th^ percentile of a chi-square with one degree of freedom), indicates that the observed frequency deviates from what is expected if there is no association. There was no evidence that different mutations are associated with differing cancer stages (p-value of 0.446). Even when analyzing only the early staged samples (I and II) yielded no significance (p-value of 0.240). Cancer type has been divided into adenocarcinoma, squamous cell carcinoma, and ‘others’, which incorporated the large cell carcinoma, pleomorphic carcinoma and carcinoid cases (miscellaneous types). A higher than expected number of *KRAS* and *EGFR* mutations were observed among adenocarcinoma cases, with fewer than expected among squamous cell and other cancer types (p-value of <0.0001) (Table 2).

**Table 2:** Statistics table of cancer type by mutation in the 833 samples.

*Cancer type*	*Mutation*							
	ALK	BRAF	EGFR	KRAS	PIK3CA	HER2	OTHERS	Total
**AD**^a^								
Frequency	1	9	**57**	**198**	5	0	**287**	557
Expected	0.67	6.02	**38.78**	**138.41**	6.69	0	**366.43**	
Chi-square	0.16	1.48	**8.56**	**25.66**	0.43	0	**17.22**	
**SQ**^b^								
Frequency	0	0	**1**	**4**	5	0	**188**	198
Expected	0.24	2.14	**13.79**	**49.2**	2.38	0	**130.26**	
Chi-square	0.24	2.14	**11.86**	**41.53**	2.88	0	**25.59**	
**OTHERS**								
Frequency	0	0	**0**	**5**	0	0	**73**	78
Expected	0.094	0.84	**5.43**	**19.38**	0.94	0	**51.31**	
Chi-square	0.094	0.84	**5.43**	**10.67**	0.94	0	**9.17**	
**Total**	1	9	58	207	10	0	548	833

There is strong evidence of an association.

^a^Adenocarcinoma.

^b^Squamous cell carcinoma.

**Table 3:** Statistics table of gender by mutation in the 833 samples.

*Gender*	*Mutation*							
	ALK	BRAF	EGFR	KRAS	PIK3CA	HER2	OTHERS	Total
**Female**								
Frequency	1	3	**42**	118	5	0	254	423
Expected	0.51	4.57	**29.45**	105.12	5.08	0	279.80	
Chi-square	0.47	0.54	**5.35**	1.58	0.0013	0	2.38	
**Male**								
Frequency	0	6	**16**	89	5	0	297	410
Expected	0.49	4.43	**28.55**	101.88	4.92	0	271.20	
Chi-square	0.49	0.56	**5.52**	1.63	0.0013	0	2.45	
**Total**	1	9	58	207	10	0	548	833

There is reasonable evidence of an association.

**Table 4:** Statistics table of smoking history by mutation in the 833 samples.

*Smoking history*	*Mutation*							
	ALK	BRAF	EGFR	KRAS	PIK3CA	HER2	OTHERS	Total
**Never**								
Frequency	0	1	**24**	7	0	0	**25**	57
Expected	0.07	0.62	**3.97**	14.16	0.68	0	**37.5**	
Chi-square	0.07	0.24	**101.06**	3.62	0.68	0	**4.17**	
**Ever**								
Frequency	1	8	**34**	200	10	0	523	776
Expected	0.93	8.38	**54.03**	192.84	9.32	0	510.5	
Chi-square	0.0053	0.017	**7.43**	0.27	0.05	0	0.31	
**Total**	1	9	58	207	10	0	548	833

There is strong evidence of an association.

A reasonable effect of gender was observed, with more females and fewer males exhibiting the *EGFR* mutation than expected (p-value of 0.00563) (Table 3). Smoking history of the patients was classified as ever smoked versus never smoked. Investigation of the type of mutation in relation to smoking history identified strong evidence of an association between individuals with the *EGFR* mutation and those who had never smoked (p-value of <0.001) (Table 4).

In certain cases, invasion of the tumour cells into different areas of the body occurred. These invasions involve the vasculature, pleura, and lymphatics. The mutation status was reasonably associated with vascular, potentially with pleural, but not with lymphatic invasion (p-values of 0.00164, 0.0482, and 0.313, respectively). In terms of vascular invasion effect, fewer individuals were observed with the *EGFR* mutation than expected.

Metastasis can also occur in the lymph nodes, which are divided into three groups based on their anatomical location in the body. N1 indicates metastasis in the ipsilateral hilar, peribronchial and intrapulmonary nodes, and N2 indicates metastasis in the ipsilateral mediastinal and subcarinal nodes. N3 indicates metastasis in the contralateral lymph nodes or supraclavicular lymph nodes, which was not found in any case of this cohort. The presence of a mutation was not associated with individuals exhibiting lymph node metastasis of N1, N2, or none (p-value of 0.864).

Survival analysis of these cases looked at the 5-year survival of the individuals. Due to the small number of cases exhibiting the *ALK*, *BRAF*, *PIK3CA* and *HER2* mutations, these cases were combined under ‘Others’. The sample size for this analysis was smaller than those used in previous analyses for a variety of reasons. Individuals diagnosed less than five years prior to analysis were excluded. Also, several individuals had either no follow-up information or insufficient information to determine their 5-year survival. Lastly, individuals with more than one mutation were regarded as one case instead of multiple. These restrictions decreased the sample size to 631 for this analysis, with 290 of these being death within 5 years. Examining presence of a mutation against 5-year survival yielded a p-value of 0.2701 indicating that the data provided no evidence of the mutations affecting 5-year survival. Analysis using Cox proportional hazards analysis also failed to show evidence of an association between mutation and overall survival. Overall, this indicates no difference in prognosis across the different gene mutations.

## Discussion

The frequencies of mutations in NSCLC, especially *EGFR* and *ALK*, in the data are lower than those found in other studies, while *KRAS* is similar to those published by Sequist et al. [8]. However, this study did not distinguish which exon mutations of each gene were analyzed for, which would suggest they only tested for the most common ones. The exact reason for the difference in results is unclear. One of the possible explanations is that in other studies, the examined tissues were mainly obtained from advanced stage lung cancers while in this study the cases were relatively early stage lung cancers. In addition, the cases reported in the literature often used small biopsy specimens whereas this study used samples from surgically resected cancer tissue, which minimizes the likelihood of sampling error. Although the statistical analysis of mutations in different cancer stages in this cohort produced non-significant results, it may be related to the sample size since most of the cases were at a relatively early stage of the disease. It is also worth noting that when observing mutation frequencies among adenocarcinoma cases, which is the most frequently observed subtype, the results were lower than those observed in previous studies except the *KRAS* mutation, which was 10% higher. These results even contradict those from a similar study, but a different cohort done at the Queen Elizabeth II Health Sciences Centre in Halifax, Canada. That study examined only adenocarcinoma cases with mixed early and later stages and observed mutation frequencies similar to those in previously published studies [8]. This implies that comparing to later stage of lung adenocarcinoma, early stage of the disease may have different frequencies of the gene mutations. However, the phenomenon was not demonstrated in this cohort itself. This is likely due to only a small percentage of the cases in this cohort having a later stage, and this smaller sample size may have influenced the results.

Previous studies have shown mixed results on whether mutation status has a predicative factor in survival rate of the patients. Johnson et al. (2013) [30] has suggested the KRAS mutation predicts a shorter survival for patients with advanced lung adenocarcinoma, however Bauml et al. (2013) [31] did not determine that EGFR and KRAS had prognostic abilities for advanced NSCLC. This potential difference could be due to the former examining adenocarcinoma samples only. The results of this study showed no correlation, similar to the latter mentioned study, however it is important to note the difference in staging. As observed, previous studies generally used populations with advanced staging, contrary to the current one. As mentioned, no correlation was observed between cancer staging and mutational status, regardless of whether the whole cohort was analyzed or only those with staging I and II. This could indicate that, for instance, the KRAS mutation is not associated with any one cancer stage but could affect mortality in those with later stages, in particular adenocarcinoma, based on previous studies.

As expected based on the literature, only a small percentage of the squamous cell carcinoma exhibited any of the mutations screened for in this study (approximately 5%) [32]. Further to this, for the cases exhibiting a *PIK3CA* mutation, five were squamous cell carcinoma and the remaining five were adenocarcinoma. This was expected as these mutations are reported to be equally as common among both cancer types [33]. This specific mutation has more commonly been observed in oral squamous cell carcinoma, involved the oral cavity, pharynx, and larynx, although typically in advanced stages. This would suggest the mutation has a larger role in tumour progression as opposed to its initiation [34].

Very strong evidence linking smoking history to mutation status was reported in the literature. Studies have shown relationships between smoking and the mutations, with a significant correlation between cigarette smoking and the *KRAS* mutation, and a higher association between females and the *KRAS* mutation [35, 36]. Neither of these correlations was observed in this study. However, the opposite trend for smoking history was observed with the *EGFR* mutation, in that if the individual harbored mutations in this receptor, there was a higher likelihood that they had never smoked. Previous studies suggest they make up a distinct subset of lung cancers [37, 38]. The “never smoking” lifestyle of the patient is not an effective predictor of whether the patient will benefit from tyrosine-kinase inhibitors however, making molecular profiling more reliable [39].

An important limitation to observe throughout this study includes the inability to evaluate all the various types of mutations found among each gene type. For instance, while this study analyzed for the *EGFR* L858R and T790M point mutations, and exon 19 deletion and exon 20 insertion, there are other types of *EGFR* mutations not included in this study. These include point mutations G719C/S/A in exon 18, V765A AND T783A in exon 20, and L861Q in exon 21, to name a few [40]. Because of the complexity of lung cancers, the most clinically relevant mutation types were chosen for this study.

## Conclusion

This study has provided an analysis of lung cancer tumours at early stages, indicating the frequency of mutations and specific correlations with patient data. Significant correlations include cancer type, gender and smoking history. As these results differ from those in previously published data, these highlight new avenues for lung cancer research as well as indicating the possibility of potential risk factors found in this particular geographical location but not others. This study provides clinically relevant data on individual mutations that will aid guiding future research in personalized medicines that will ultimately improve lung cancer survivorship and quality of life.
